# Long-term clinical outcomes in patients with viral hepatitis related liver cirrhosis after transjugular intrahepatic portosystemic shunt treatment

**DOI:** 10.1186/s12985-018-1067-7

**Published:** 2018-10-01

**Authors:** Dengke Teng, Hao Zuo, Lin Liu, Jinghui Dong, Lei Ding

**Affiliations:** 1 Department of Ultrasound, China-Japan Union Hospital of Jilin University, Changchun, 130022 Jilin Province China; 2 Department of Pain, The Second Hospital of Jilin University, Changchun, 130041 Jilin Province China; 3 Department of Radiology, China-Japan Union Hospital of Jilin University, 126 Xintai St, Changchun, 130022 Jilin Province China; 4 Department of Radiology, 302 Military Hospital of China, 100 West Fourth Ring Rd, Beijing, 100039 China

**Keywords:** Transjugular intrahepatic portosystemic shunt, Liver cirrhosis, Portal hypertension, Refractory ascites, Variceal bleeding

## Abstract

**Background:**

Transjugular intrahepatic portosystemic shunt (TIPS) procedure has played a vital role in management of portal hypertension. Thus, we aimed to investigate the natural history, long-term clinical outcome, predictors of survival in viral hepatitis related cirrhotic patients post-TIPS.

**Method:**

A total of 704 patients with complete followed-up data were enrolled, and clinical characteristics of patients were collected and analyzed. Kaplan-Meier method was used to calculate survival, and comparisons were made by log rank test. A multivariate analysis of factors influencing survival was carried out using the Cox proportional hazards regression model.

**Results:**

TIPS implatantion significantly decreased portal vein pressure with 9.77 cmH_2_O reduction, without influencing long-term liver functions. The total incidence rate of major complication post-TIPS, including HE and re-bleeding/bleeding, was 37.9% and 15.5%, respectively. Patients in Child-Pugh C stage revealed higher overt hepatic encephalopathy (HE) occurrence (65.6%), while patients receiving covered, 6 mm in diameter stents indicated notably lower incidence of HE in comparison with other groups (6.4%). The median survival was > 60 months, 27.0 months, and 11.5 months in cirrhotic patients with variceal bleeding, refractory ascites, and both complications, respectively. The cumulative 5-year survival was significantly higher in patients with variceal bleeding (75.6%) in comparison with either that in patients with refractory ascites (12.5%) or that in patients with both complications (1.96%) (*P* < 0.0001). Covered stents usage, baseline model for end-stage liver disease (MELD) score, and baseline Child-Pugh classification were predictive of survival (*P* < 0.001). Other variables including age, male gender, and pre-TIPS PVP were not emerged as significant predictors (*P* > 0.05).

**Conclusion:**

TIPS was an effective and safe therapeutic method for decompression of portal hypertension and for treatment of its complications. Careful selection of patients with minimal liver dysfunction for TIPS implantation was essential for better long-term outcomes.

## Background

Portal hypertension is one of the most common problems among patients with liver cirrhosis, and therapeutic approaches of the complications are still the challenging tasks [1, 2]. Development of portal hypertension always results in the formation of collateral circulation in portal vein system, which leads to the portal venous flow into systemic circulation and directly increase the incidence of several clinical consequences, e.g. variceal bleeding and refractory ascites [3–7]. Although a variety of treatment methods have been built up, controversy remains as to the most effective therapeutic algorithm for the complications of portal hypertension [8, 9].

Transjugular intrahepatic portosystemic shunt (TIPS) surgery inserted metal stent into the liver parenchyma radiologically to establish a shunt between portal vein and hepatic vein/inferior vena cava. It is an efficient method for reducing portal pressure, and has been widely used for treatment of complications of portal hypertension [10, 11]. TIPS has gradually become the first-line therapeutic choice for cirrhotic patients with acute variceal hemorrhage who failed with endoscopic hemostasis [12, 13], with an estimated technical successful rate of 93–100% [2]. TIPS is also used in treatment of refractory ascites [14–16] and hepatorenal syndrome [17] due to the circulatory effects on portal hypertension. However, there have been concerns about TIPS implantation, especially with high rate of hepatic encephalopathy (HE) post-TIPS [14]. More recently, the development and usage of covered metal stents significantly reduce the shunt dysfunction in comparison with bare mental stents insertion [18], leading to the lower occlusion rate of consecutive bleeding and improvement of overall survival [19, 20]. However, few studies focused on the long-term outcomes of patients receiving TIPS for complications of portal hypertension and liver cirrhosis, especially with respect to variceal bleeding versus refractory ascites. Thus, in this retrospective study, we evaluated the long-term efficacy and outcomes of TIPS in treatment of variceal bleeding and/or refractory ascites. The major objectives of the present study were to observe the occurrence of clinical complications of TIPS, and predictors of survival.

## Methods

### Patients and followed-up

We screened integrated database which included a total of 1024 patients with viral hepatitis related liver cirrhosis who underwent TIPS insertion between June 2004 and December 2012 in China-Japan Union Hospital and 302 Military Hospital. The indication for TIPS treatment included acute or recurrent variceal bleeding and refractory ascites. TIPS insertion was technically not feasible in 89 patients, including 46 patients with unsuccessful portal vein puncture, 28 patients with portal vein malformation, and 15 patients with portal vein thrombosis. There were 231 cases who were lost to follow-up after TIPS insertion. Thus, eventually 704 patients with complete 5-year followed-up data or confirmed death within 5-year followed-up period were enrolled in this study. The TIPS procedures were accomplished by different specialists followed with the same protocol. Anticoagulant drugs, ornithine aspartate, and lactulose were routinely used after TIPS insertion. All patients were treated for primary diseases in the followed-up period, such as nucleos(t)ide analogue therapy for HBV infection. Followed-up data were obtained by in-patients/out-patients visit, or telephone calls every year. Biochemical and ultrasound assessments were performed as routine examination. The study protocol was approved by Ethics Committees of both China-Japan Union Hospital and 302 Military Hospital on December 2016, and data were collected on January and February 2017.

### Assessment of clinical characteristics

TIPS procedures were conducted using standard techniques [21]. Serum biochemical assessments (including alanine aminotransferase [ALT], aspartate aminotransferase [AST], albumin [ALB], total bilirubin [T-BIL], blood urea nitrogen [BUN], and serum creatinine [Cr]) were measured using an automatic analyzer (Hitachi 7170A, Hitachi Ltd., Tokyo, Japan). Coagulation function (including prothrombin time [PT], thrombin time [TT], activated partial thromboplastin time [APTT], fibrinogen [Fib], prothrombin activity [PTA], and international normalized ratio [INR]) were measured using a coagulation analyzer (PUN-2048, Perlong Medical Products, Beijing, China). Abdominal ultrasound examination was measured using a Doppler ultrasound diagnostic apparatus (NemioXG, Toshiba, Tokyo, Japan). The stiffness of liver was measured using FibroScan 502 (Echosens, Pairs, France). The severity of liver disease was assessed using traditional Child-Pugh classification as described previously [22, 23]. T-BIL, ALB, INR, ascites, and HE grade was involved for Child-Pugh scoring.

### Statistical analysis

All data were analyzed using SPSS version 19.0 for Windows Software (SPSS Inc., Chicago, IL, USA). Wilcoxon’s matched pairs test or Dunn’s multiple comparison test were used for comparison of quantitative data. Chi-squared test was used for comparison of categorical data. Kaplan-Meier method was employed to calculate survival from the time of TIPS treatment, and comparisons were made by log rank test. A multivariate analysis of factors influencing survival was carried out using the Cox proportional hazards regression model. The potential predictor variables for survival was age, male gender, and pre-TIPS PVP, complications, stents usage, model for end-stage liver disease (MELD) score, and Child-Pugh classification. We firstly perform univariate analysis for each variable and then perform multivariate analysis using all the variables. All tests were two-tailed, and *P* values of less than 0.05 were considered to indicate significant differences.

## Results

### Baseline characteristics of enrolled patients

The retrospective cohort comprised 704 of liver cirrhotic patients with TIPS insertion. Baseline characteristics of enrolled patients were listed in Table 1. Of these patients, 581 (82.5%) showed variceal bleeding, 72 (10.2%) revealed refractory ascites, whereas 51 (7.3%) demonstrated both variceal bleeding and refractory ascites. Five hundred and eleven (72.6%) patients were male and 193 (27.4%) were female, with a mean age of 53.2 years.

**Table 1:** Baseline clinical characteristics of enrolled patients

Characteristic	Value
Patients enrolled *(n)*	704
Variceal bleeding	581
Refractory ascites	72
Variceal bleeding and refractory ascites	51
Age, *years*, *(mean ± SD)*	53.2 ± 13.6
Gender, male/female, *(n)*	511/193
Cause of cirrhosis *(n)*	
HBV	509
HCV	134
HBV + HCV	52
HBV + HDV	9
Child-Pugh classification *(n)*	
A	219
B	421
C	64
MELD score, *(mean ± SD)*	12.8 ± 5.1
Stiffness of liver, *KPa, (mean ± SD)*	18.6 ± 10.7
Diameter of portal vein, *cm*, *(mean ± SD)*	1.52 ± 0.37
Diameter of splenic vein, *cm*, *(mean ± SD)*	0.96 ± 0.21

*MELD* model for end-stage liver disease

### TIPS insertion significantly reduced PVP of patients with liver cirrhosis, but not ameliorated liver functions

In all 704 enrolled patients with TIPS treatment, direct measurements of portal vein pressure (PVP) were carried out in 487 cases before and after stent insertion. TIPS insertion significantly decreased PVP with 9.77 cmH_2_O reduction (36.81 ± 8.68 cmH_2_O vs. 27.04 ± 7.79 cmH_2_O, Wilcoxon’s matched pairs test, *P* < 0.0001). All patients were also received anti-fibrosis and anti-primary diseases therapies, and liver functions were assessed in each visit. There were no remarkable differences in ALT, T-BIL, albumin, and stiffness of liver after TIPS insertion in comparison with baseline (Dunn’s multiple comparison test, *P* > 0.05, Table 2). All patients were routinely treated with anticoagulant drugs at a weight-dependent dose for 1 year after TIPS insertion, leading to the disturbance of blood coagulation which presented as the reduction in PTA and elevation in INR (Dunn’s multiple comparison test, *P* < 0.05 compared with baseline, Table 2). However, blood ammonia was also increased, especially in the early stage after TIPS implantation, although ornithine aspartate were routinely used (Dunn’s multiple comparison test, *P* < 0.05 compared with baseline, Table 2).

**Table 2:** Changes in liver functions of liver cirrhotic patients with TIPS treatment

	Baseline	1 year	2 years	3 years	4 years	5 years
	(*n* = 704)	(*n* = 638)	(*n* = 587)	(*n* = 530)	(*n* = 485)	(*n* = 445)
ALT (U/L)	51 ± 28	47 ± 19	55 ± 31	52 ± 21	58 ± 20	55 ± 18
T-BIL (μmol/L)	29.2 ± 19.7	34.8 ± 20.1	32.9 ± 21.8	35.9 ± 20.8	34.6 ± 19.7	27.6 ± 18.0
Albumin (g/L)	28.9 ± 10.2	29.1 ± 11.7	30.0 ± 9.8	28.7 ± 8.2	29.7 ± 9.2	30.1 ± 12.8
Blood ammonia (μmol/L)	64.7 ± 25.9	87.6 ± 27.1 ^##^	81.5 ± 19.8 ^##^	76.2 ± 20.5 ^##^	78.2 ± 21.2 ^#^	71.0 ± 11.6
PTA (%)	56.8 ± 12.5	37.8 ± 9.1 ^#^	51.4 ± 8.7	49.2 ± 11.0	50.1 ± 10.2	50.8 ± 8.9
INR	1.36 ± 0.23	1.90 ± 0.46 ^#^	1.48 ± 0.51	1.50 ± 0.49	1.50 ± 0.44	1.47 ± 0.38
Stiffness (KPa)	18.6 ± 10.7	17.6 ± 9.8	17.9 ± 12.7	16.8 ± 8.6	16.7 ± 9.1	17.1 ± 10.2

^#^*P* < 0.05, ^##^ *P* < 0.01 compared with baseline, Dunn’s multiple comparison test

### Complications after TIPS implantation

The major complications after TIPS therapy included HE and re-bleeding/bleeding. The total incident rate of HE and re-bleeding/bleeding was 37.9% and 15.5%, respectively. Patients with variceal bleeding and refractory ascites indicated higher incidences for both HE and re-bleeding/bleeding (Chi-squared test, *P* < 0.05, Table 3). Patients with Child-Pugh C revealed a significant elevated incidence of HE than those with Child-Pugh A or B (Chi-squared test, *P* < 0.01, Table 3). However, there were no remarkable difference in the incidence of re-bleeding/bleeding among Child-Pugh classification (Chi-squared test, *P* > 0.05, Table 3). Moreover, uncovered (*n* = 130) and covered (*n* = 574) metal stents were used for portosystemic shunt. No significant incidence of HE and re-bleeding/bleeding were found between patients using uncovered and covered stents (Chi-squared test, *P* > 0.05, Table 3). However, patients receiving covered, 6 mm in diameter stents indicated notably lower incidence of HE in comparison with other groups (Chi-squared test, *P* < 0.0001, Table 3).

**Table 3:** The incidence of major complications after TIPS treatment

	HE	Re-bleeding/Bleeding
Total	267/704 (37.9%)	109/704 (15.5%)
Variceal bleeding	208/581 (35.8%)	81/581 (13.9%)
Refractory ascites	31/72 (43.1%)	6/72 (8.3%)
Variceal bleeding and refractory ascites	28/51 (54.9%) ^#^	22/51 (43.1%) ^##^
Child-Pugh classification		
A	83/219 (37.8%)	37/219 (16.9%)
B	142/421 (33.7%)	61/421 (14.5%)
C	42/64 (65.6%) ^##^	11/64 (17.2%)
Type of stent		
Uncovered stent (*n* = 130)	56/130 (43.1%)	17/130 (13.1%)
Diameter of stent = 8 mm (*n* = 47)	17/47 (36.1%)	5/47 (10.6%)
Diameter of stent = 10 mm (*n* = 83)	39/83 (47.0%)	12/83 (14.5%)
Covered stent (*n* = 574)	211/574 (36.8%)	92/574 (16.0%)
Diameter of stent = 6 mm (*n* = 94)	6/94 (6.4%) ^###^	14/94 (14.9%)
Diameter of stent = 7 mm (*n* = 178)	67/178 (37.6%)	31/178 (17.4%)
Diameter of stent = 8 mm (*n* = 302)	138/302 (45.7%)	47/302 (15.6%)

^#^*P* < 0.05, ^##^ *P* < 0.01, and ^###^ *P* < 0.01 compared with other groups, Chi-squared test

### Patients survival

**Fig. 1:**
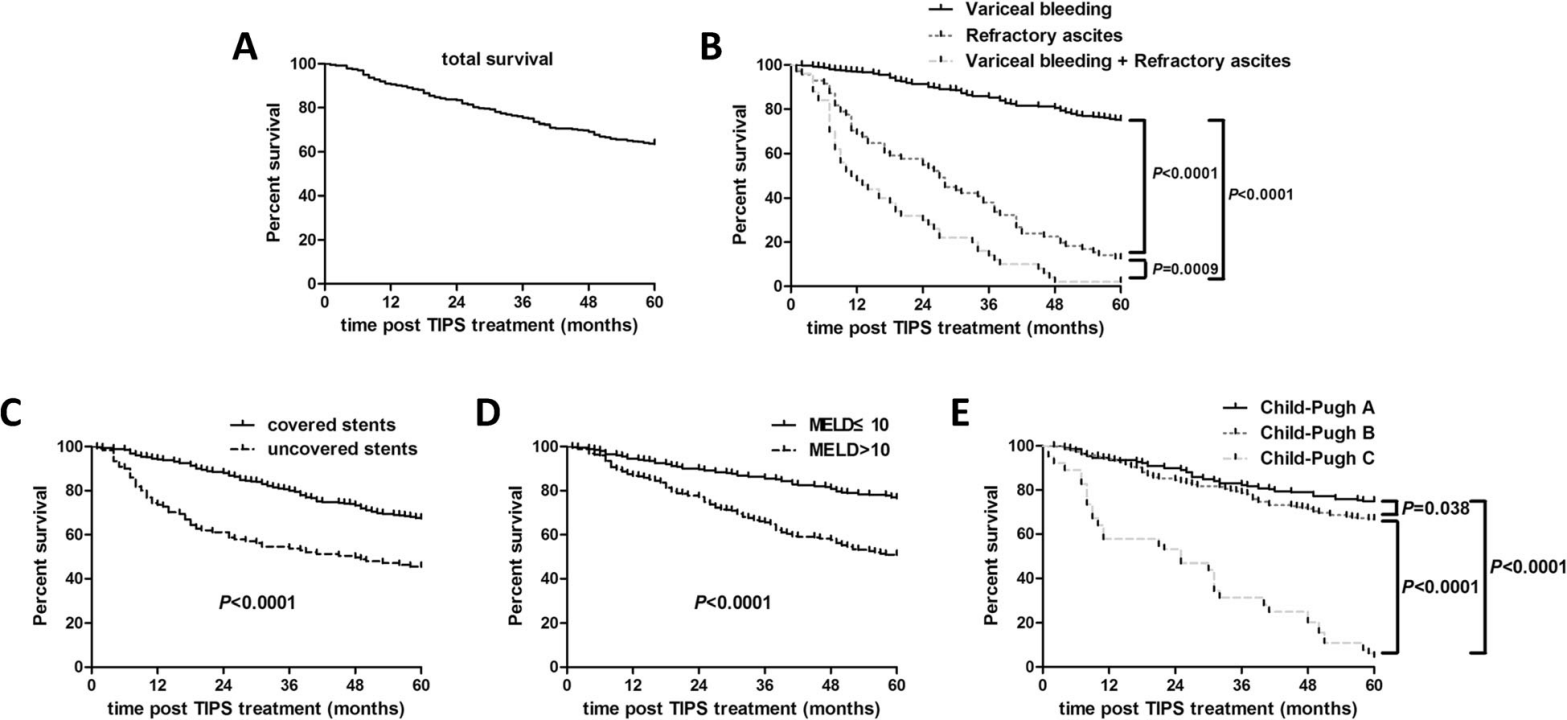
Kaplan-Meier survival analysis of patients after transjugular intrahepatic portosystemic shunt (TIPS) implantation. **a** Overall survival. **b** Comparison of probability of survival among patients with variceal bleeding and/or refractory ascites. **c** Comparison of probability of survival between patients with uncovered and covered stent. **d** Comparison of probability of survival between patients with baseline MELD score ≤ 10 and > 10. **e** Comparison of probability of survival among patients with different Child-Pugh stage

Overall median survival was > 60 months with the cumulative 5-year survival of patients in 63.6% (Fig. 1a). The median survival was > 60 months, 27.0 months, and 11.5 months in cirrhotic patients with variceal bleeding, refractory ascites, and both complications, respectively. Moreover, the cumulative 5-year survival was significantly higher in patients with variceal bleeding (75.6%) in comparison with either that in patients with refractory ascites (12.5%, hazard ratio [HR] = 69.28 [95% CI 39.01–123.0], *P* < 0.0001, Fig. 1b) or that in patients with both complications (1.96%, HR = 0.00025 [95% CI 0.00011–0.00059], *P* < 0.0001, Fig. 1b). On multivariate analysis covered stents usage (HR = 2.96 [95% CI 2.06–4.25], *P* < 0.0001, Fig. 1c), baseline MELD score (HR = 0.40 [95% CI 0.31–0.41], *P* < 0.0001, Fig. 1d), and baseline Child-Pugh classification (Child-Pugh A versus Child-Pugh B, HR = 0.73 [95% CI 0.54–0.98], *P* = 0.038; Child-Pugh A versus Child-Pugh C, HR = 0.034 [95% CI 0.019–0.058], *P* < 0.0001; Child-Pugh B versus Child-Pugh C, HR = 0.038 [95% CI 0.023–0.064], *P* < 0.0001; Fig. 1e) were predictive of survival. Other variables included in the final Cox proportional hazards model were age, male gender, and pre-TIPS PVP, but none emerged as significant predictors (*P* > 0.05).

## Discussion

To the best of our knowledge, the current study represented one of the largest cohort of patients who received TIPS as therapeutic method for the complications of portal hypertension and liver cirrhosis, and followed-up for the longest period of time, thereby allowing us to better understand plenty of issues related to TIPS implantation for variceal bleeding and/or refractory ascites.

In this retrospective study, shunt insertion was successful in 91.3% (935/1024) of patients scheduled. The baseline characteristics revealed that distribution of underlying diseases was typical for China, with chronic viral hepatitis as the major causation for liver cirrhosis [24]. In agreement with previous findings [25–28], we confirmed that TIPS is effective as treatment for variceal bleeding and refractory ascites in cirrhotic patients without improvement of liver function. TIPS implantation could decrease the portal pressure gradient by 20–50% of the initial pressure, and maintained under 12 mmHg [29, 30]. This was consistent with the current results of reduction in direct measurement of PVP. However, there were no remarkable differences in the degree of reduction of PVP post-TIPS insertion among patients with variceal bleeding and/or refractory ascites. Although the initial reduction in PVP after TIPS insertion was considered to be a predictor for rebleeding risk, but not for survival [31], we did not find notable correlation between baseline/reduction of PVP and survival, indicating that PVG might not be the predictor for survival post-TIPS treatment.

In consistent with Membreno et al. [32] and Heinzow et al. [2], the current results demonstrated that overall long-term survival was significantly better in patients with TIPS due to variceal bleeding (> 60 months) than that in patients with TIPS due to refractory ascites (27.0 months), while the combination of both complications further worsened the survival (11.5 months, *P* < 0.001) with higher rate of major complications. The overall occurrence of HE was nearly 40% which was higher than previous reports, although we routinely prescribed ornithine aspartate and lactulose to all patients post-TIPS. The use of smaller diameter stents might be associated with lower risk of HE post-TIPS, as the development of refractory HE requiring reduction in shunt diameter in 8–10% of patients [33]. This was in accordance with our results showing the lower HE occurrence in patients with 6 mm-diameter of stent insertion, although elevated risk of treatment failure was also observed among patients with smaller 8-mm stent from a randomized controlled trials [34].

TIPS implantation increased the risk of acute liver and/or cardiac decompensation and failure. Thus, careful selection of patients with liver cirrhosis and portal hypertension was crucial to the successful outcome post-TIPS [35, 36]. Previous study revealed that better liver function might respond better to TIPS insertion [37]. The baseline age < 55 years, T-BIL < 35 μmol/L, and serum sodium > 135 mmol/L indicated beneficial survival post-TIPS. Original Child-Pugh stage [2], modified Child-Na score with serum sodium incorporation [38], and MELD score [39] were independent prognostic factor of survival. We showed that patients in Child-Pugh C stage demonstrated higher incidence of overt HE. Moreover, in agreement with previous findings, Child-Pugh stage and MELD score were significant indicator for survival post-TIPS, probably due to the fact that both scoring systems were validated tools for assessing prognosis [40]. Thus, approximate 60% of overall 5-year survival was not surprising as the enrolled patients had minimal liver dysfunction at baseline.

Uncovered metal stents were one of the treatment choice for establishing TIPS tracts [41], with approximate 20% use of all TIPS procedures in United States [42]. The higher rate of shunt dysfunction with consecutive bleeding complications [43] has been largely overcome after the development of covered metal stents [18, 19, 44]. Although Bureau and colleagues did not detect survival benefit of covered and bare stents [45], more recently meta-analyses of randomized controlled trials revealed that covered stents for TIPS improved overall survival [20], especially in prevention of variceal re-bleeding [46]. In addition, it was also reported that 1-year probability of remaining free of HE in patients with post-covered TIPS was numerically lower than that with bare stents [35, 47]. In the present study, we found that there were no remarkable differences in the occurrences of either HE or re-bleeding between covered and uncovered stents. Furthermore, the use of covered stents was predictive of beneficial survival, which was similar to the findings by Tan et al. in refractory ascites [35]. This was partly due to the better baseline liver function in covered stents groups, which was also found in Tan’s study [35]. Moreover, since most covered stents were used in recent years, the improvement in procedure-related skills with primary patency up to 90% within first year application also accounted for superiority of covered stents [19, 35]. Thus, as expected, the improved survival was due to era effect rather than type of stents [35], which further deepened the understanding of patients selection for TIPS implantation.

There were some limitations in this study. First, we conducted a retrospective study with limited patients numbers and no control group was established. Thus, large-scale, random control studies were needed to confirm the current results. Second, we tried to identify predictors for liver failure in patients post-TIPS. However, the definition for liver failure was hard. The classical definition for liver failure contains jaundice (T-BIL > 170 μmol/L) and coagulopathy (PTA < 40%). The use of anticoagulant drugs down-regulated PTA level, which made it hard for definition. Furthermore, only few patients suffered with high jaundice post-TIPS. Thus, we did not analyze predictors for liver failure post-TIPS. Third, the factors regarding the cause of cirrhosis were lacking in survival analysis after TIPS. Fourth, the cause of death of patients did not analyzed in the current study.

## Conclusion

TIPS was an effective and safe therapeutic method for decompression of portal hypertension and for treatment of its complications. Child-Pugh stage and MELD score were independent predictors of survival in patients with TIPS implantation. Thus, careful selection of patients for TIPS was essential for better long-term outcomes.

## Abbreviations

ALT: Alanine aminotransferase
APTT: Activated partial thromboplastin time
AST: Aspartate aminotransferas
BUN: Blood urea nitrogen
Cr: Creatinine
Fib: Fibrinogen
HE: Hepatic encephalopathy
HR: Hazard ratio
INR: International normalized ratio
MELD: Model for end-stage liver disease
PT: Prothrombin time
PTA: Prothrombin activity
PVP: Portal vein pressure
T-BIL: Total bilirubin
TIPS: Transjugular intrahepatic portosystemic shunt
TT: Thrombin time
